# Dendritic cell-associated B7-H3 suppresses the production of autoantibodies and renal inflammation in a mouse model of systemic lupus erythematosus

**DOI:** 10.1038/s41419-019-1623-0

**Published:** 2019-05-21

**Authors:** Xu Zheng, Ze Xiu Xiao, Li Hu, Xuan Fang, Liqun Luo, Lieping Chen

**Affiliations:** 1 Department of Rheumatology & Immunology, The First Affiliated Hospital of USTC, Division of Life Sciences and Medicine, University of Science and Technology of China, Hefei, AH P. R. China; 2 Immunotherapy Laboratory, Sun Yat-Sen University, Guangzhou, GD P. R. China; 3 Immunotherapy Institute, Fujian Medical University, Fuzhou, FJ P. R. China; 4 Department of Immunobiology, Yale University, New Haven, CT USA

**Keywords:** Autoimmunity, Immunotherapy

## Abstract

B7-H3 immune modulatory molecule has been implicated in the generation and pathogenesis of autoimmune diseases, the mechanism of action is less known. We explored the role of B7-H3 in the induction of autoantibodies and organ-directed inflammation in a murine systemic lupus erythematosus (SLE) model in which the immunization with DNA extracted from activated T cells induced the production of anti-DNA autoantibodies and subsequent glomerulonephritis, two hallmarks of human SLE. Mice deficient of B7-H3 or treated with a B7-H3 specific antibody produced significantly higher levels of anti-DNA autoantibodies and more severe glomerulonephritis than wild-type mice, indicating an inhibitory function of B7-H3 in this model. Interestingly, immunization of mice with DNA-pulsed dendritic cells induced severe SLE symptoms while B7-H3 on dendritic cells is required in this process. Importantly, treatment of mice with recombinant B7-H3Ig fusion protein effectively ameliorated progression of murine SLE, accompanied with decreased level of anti-DNA autoantibodies and alleviated glomerulonephritis, decreased autoantibody deposition and complement deposition in kidney. Our findings implicate a potential role of B7-H3 on dendritic cells in the induction of SLE and as a potential target for the treatment of autoimmune diseases.

## Introduction

B7-H3, a cell surface molecule of the extended CD28/B7 family, was discovered in our laboratory in 2001 by searching databases for molecules with homology to previously identified B7 molecules^1^. B7-H3 has single IgV- and IgC-like domains (2Ig form) with a transmembrane and intracellular tail in human, mice and other species^2^. In human, a unique isoform with dual IgV- and IgC-like domains (4Ig form) was also identified^3^. B7-H3 mRNA is broadly detected in normal tissues and its cell surface expression, although much rare, could also be found on activated DCs in lymphoid tissue^4,5^. While immunological function of B7-H3 is yet to be elucidated, early studies indicate that B7-H3 co-stimulates human and murine T cell proliferation and cytokine secretion in vitro and enhances tumor rejection by over-expression in tumor lines in animal models^1,6,7^. Whereas these studies suggest a positive role of B7-H3 in the regulation of T cell responses, other studies, however, indicate that B7-H3 also have inhibitory functions on several T cell responses, including inhibition of T cell proliferation in vitro and allergen-induced autoimmunity in murine models^8^. Currently there are several possible interpretations for these seeming contradictory roles of B7-H3 in immune responses. A hypothesis for these seemingly contradictory data is that differential role of B7-H3 in the regulation of distinct T cell subsets^9^. Another possible explanation is the engagement of different putative receptors by B7-H3.

Systemic lupus erythematosus (SLE) is a systemic autoimmune disease and is characterized by the presence of hyperactive immune cells and aberrant antibody responses to nuclear and cytoplasmic antigens, including characteristic anti-double-stranded DNA antibodies (anti-dsDNA Abs)^10–12^. Anti-dsDNA Abs are thought to be diagnostic markers in SLE and their presence in humans and mice often correlates with disease pathogenesis^13–16^. Furthermore, these Abs also contribute to disease progression of SLE, as indicated by glomerulonephritis, an inflammation largely due to deposition of antigen (Ag)-Abs complex and subsequent activation of complement. Qiao et al.^17^ showed that mice immunized with activated lymphocyte-derived DNA (ALD-DNA) produced high levels of anti-dsDNA Abs, and subsequently developed SLE-like syndrome, including aggravated glomerulonephritis, increased autoantibody and complement deposition. These observations resemble closely human SLE and this may provide a good model for studying human SLE pathogenesis.

Dendritic cells (DCs) are showed to be functionally abnormal in human SLE, including a reduced number of circulating conventional DCs, but increased plasmatoid DC (pDCs)^18^. In SLE, conventional DCs promote autoreactivity rather than tolerance^19^. In turn, activated T cells also promote increased type I interferon (IFN) production by pDCs^20^. Conventional DCs were also shown to contribute to the development of lupus nephritis in a mouse model^21^. Collectively, these findings implicate possible contribution of DCs to the disease progression of SLE. B7-H3 is not expressed in significant amounts on freshly isolated lymphocytes but could be induced on DCs and monocytes/macrophages upon activation^22^. Expression of B7-H3 on DCs could be further enhanced by the TH1 cytokine IFN-γ or LPS^5^. The function of B7-H3 on DCs, however, is unclear.

In this study, we show that mouse B7-H3 on DCs has a negative regulatory function for CD4+ T cell-dependent production of anti-dsDNA Abs and contributes to disease progression in a murine SLE model.

## Materials and methods

### Mice strains and cell lines

Female C57BL/6 (B6) mice, littermate control mice and B7-H3 knockout (KO) mice^9^ were used in aged 6–10 weeks. B7-H3KO mice were generated in Chen’s laboratory and have been backcrossed to B6 background for 10 generations. B6 lpr/lpr were purchased from Model Animal Research Center of Nanjing University. B6 lpr/lpr × B7-H3KO (B6 lpr/lpr-KO) mice were obtained by backcrossing between B6 lpr/lpr and B7-H3KO and all mice were housed in a specific pathogen-free (SPF) room. Mouse housing procedures were conducted according to the Guide for the Care and Use of Medical Laboratory Animals (Ministry of Health, PRC, 1998). The DC2.4 dendritic cell line (H-2^b^) originated from a B6 mouse was developed by superinfecting GM-CSF-transduced bone marrow cells with myc and raf oncogenes^23^. For cell transfection, 293T cells were seeded at 8 × 10^6^ on 10 cm dish and transfected at next day when they were 70–80% confluency. Prior to transfection, the culture medium was changed with DMEM. Mix with 30ug plasmid B7-H3Ig or control Flag-Ig and 90ug PEI incubating for 15 min in Opti-MEM. After adding the complexes to the dishes for 5–6 days, supernatant was collected for further purification.

### Detection of anti-DNA autoantibodies

IgG antibodies to ALD-DNA were assessed using a previously described enzyme-linked immunosorbent assay (ELISA) with modification^24^. Briefly, 96-well plates were coated with double stranded salmon sperm DNA 100 μg/well at 100 μg/ml and placed overnight at 4 ℃. The plates were then washed four times with PBS containing 0.05% Tween-20, dried and were added 200 μl of blocking solution (PBS containing 10% FBS) per well. Mouse serum diluted 1:50 in PBS containing 1% BSA was added to each well and incubated for 2 h at 37 ℃. The plates were washed 10 times, followed by the addition of anti-mouseIgG1-Fc-HRP (horseradish peroxidase) specific secondary antibodies which were diluted 1:4000 in PBS containing 1% FBS 100 μl/well. After extensive washing, the plates were incubated for 20 min with 100 μl/well TMB. The absorbance was measured at 450 nm on a microtiter plate reader (Southern Biotech, USA).

### Flow cytometry analysis and antibodies

The monoclonal antibody (mAb) clone 14 M is a mouse anti-mouse IgG1 mAb against B7-H3 generated by the immunization of a B7-H3KO mouse. PE anti-mouse CD8a, PE-cy7 or APCanti-mouse CD4, FITC anti-mouse CD3, PE anti-mouse CD11c and isotype-matched mAbs were purchased from BD Biosciences; and anti-p-STAT3 antibody was purchased from Cell Signaling Technology. Fixation/Permeabilization Kit were purchased from eBioscience. For cell surface staining, cells were directly stained with either IgG control or fluorescence-conjugated antibodies. For intracellular staining, cells were permeabilized and fixed after surface staining, and stained with fluorescence-conjugated antibodies or IgG controls.

### DNA immunization

Genomic DNA was extracted from Con A-stimulated splenocytes (ALD-DNA) as described previously^25^. The concentration of ALD-DNA was determined by the absorbance (A) at 260 nm, and the final A260/280 was >1.8. All procedures were practiced under aseptic conditions. Mice were subcutaneously (sc) injected under the dorsal skin with ALD-DNA (50 μg/mice) in complete Freund’s adjuvant (sigma), followed by two booster immunizations (50 μg/mice) emulsified with incomplete Freund’s adjuvant at weeks 2 and 4^26^. Mice were intraperitoneally (i.p.) injected with 14 M or control Ig. Mice were bled from retro-orbital sinus before immunization and every 2 weeks after their initial immunization and sera were prepared for the assay. All mice were sacrificed 12 weeks post-immunization, spleen, lymph nodes and kidneys were collected for further analysis.

### Immunization by bone marrow-derived dendritic cell (BMDC)

DCs were prepared from mouse bone marrow. Briefly, bone marrow cells were flushed from femur and tibia. The harvested cells were cultured in RPMI-1640 complete medium with 10% fetal bovine serum (Gibco) containing 50 ng/ml recombinant mouse (rm) GM-CSF (peProTech), 12.5 ng/ml rmIL-4(peProTech). At day 3, fresh medium containing rmGM-CSF and rmIL-4 was added to each culture flask. At day 6, half of the cell suspension was centrifuged, suspended in the same volume of cell culture medium and returned to the culture flask. The cells were harvested and used for experiments. For immunization, BMDC were incubated with 5ug/ml ALD-DNA overnight. After extensive wash, 5 × 10^5^ BMDC-ALD-DNA were intravenously (i.v.) injected, followed by i.p. injection with 14 M or control Ig. Mice were bled from retro-orbital sinus every 2 weeks and sacrificed at week 10 and spleen, lymph nodes and kidneys were collected for further analysis. For early stage treatment, 5 × 10^5^ BMDC-ALD-DNA were i.v. injected, followed by i.p. injection with B7-H3Ig or control Ig at week 0. For late stage treatment, mice were treated with B7-H3Ig or control Ig at week 4 after the immunization with BMDC-ALD-DNA.

### Pathological assessment and evaluation of renal histopathology

Kidneys from ALD-DNA-immunized mice were frozen in dry ice, embedded with OCT medium and 5 μm sections were obtained and stained with hematoxylin (HE). Kidney sections assessed by a single observer, grading the kidneys for glomerular inflammation, proliferation, crescent formation, and necrosis. Interstitial changes and vasculitis were also noted. Scores from 0 to 3 were assigned for each of these features and then added together to yield a final renal scores. Glomerular inflammation was graded: 0, normal; 1, few inflammatory cells; 2, moderate inflammation; and 3, severe inflammation. Detailed pathological assessment was performed as described previously^27^.

Sections were stained with HRP-conjugated goat anti-mouse IgG (Sigma) or HRP-conjugated rabbit anti-mouse C3 (Sigma). Tissues from mouse kidneys were prepared and stained with HE and eosin using standard procedures.

### Cytometric bead array (CBA)

The 14 M or control Ig at 5 μg/ml was aspirated into the 12-well plates overnight. After washing, 5 × 10^5^ DC2.4 cells were added into the 12-well plates. Supernatant was collected 24 h later, and tested with mouse inflammation kit (BD). For the inflammation cytokine CBA assay, 50 μL of supernatant were stained with the mixture of mouse cytokine capture bead suspension and the PE detection reagent. After 2 h of incubation, samples were washed and then analyzed by using the BD CBA software. Mouse inflammation cytokine standards provided with the kit were diluted and used in parallel to samples for preparation of the standard curves as instructed.

### Quantitative real-time PCR (qRT-PCR) assay

Total RNA was extracted using RNeasy (Qiagen) and was reverse transcribed to cDNA using Taqman reverse transcription reagents (Applied Biosystems) according to the manufacturer’s instructions. Primer and probe sets were obtained from Applied Biosystems. qRT-PCR was performed using the Taqman Universal PCR Master Mix. Primers used to amplify specific gene fragments as follow: B7-H3: 5′GACACGGATGCCACCCTACGCTG (forward) and 5′CTGTGATGGTGACTGAGCCGTGAG (reverse) and HPRT: 5′TCAACGGGGGACATAAAAGT (forward) and 5′TGCATTGTTTTACCAGTGTCAA (reverse). Expressions of B7-H3 were calculated by their ratios to HPRT.

### Statistical analysis

The data are expressed as mean ± standard deviation (SD). Two-tailed student’s tests were used to calculate statistical significance. Data were shown as a representative experiment of three independent experiments. Statistical significance was defined as *P < 0.05, **P < 0.01, and ***P < 0.001.

## Results

### Intrinsic B7-H3 suppresses the production of anti-DNA Abs and renal inflammation in a SLE mouse model

**Fig. 1:**
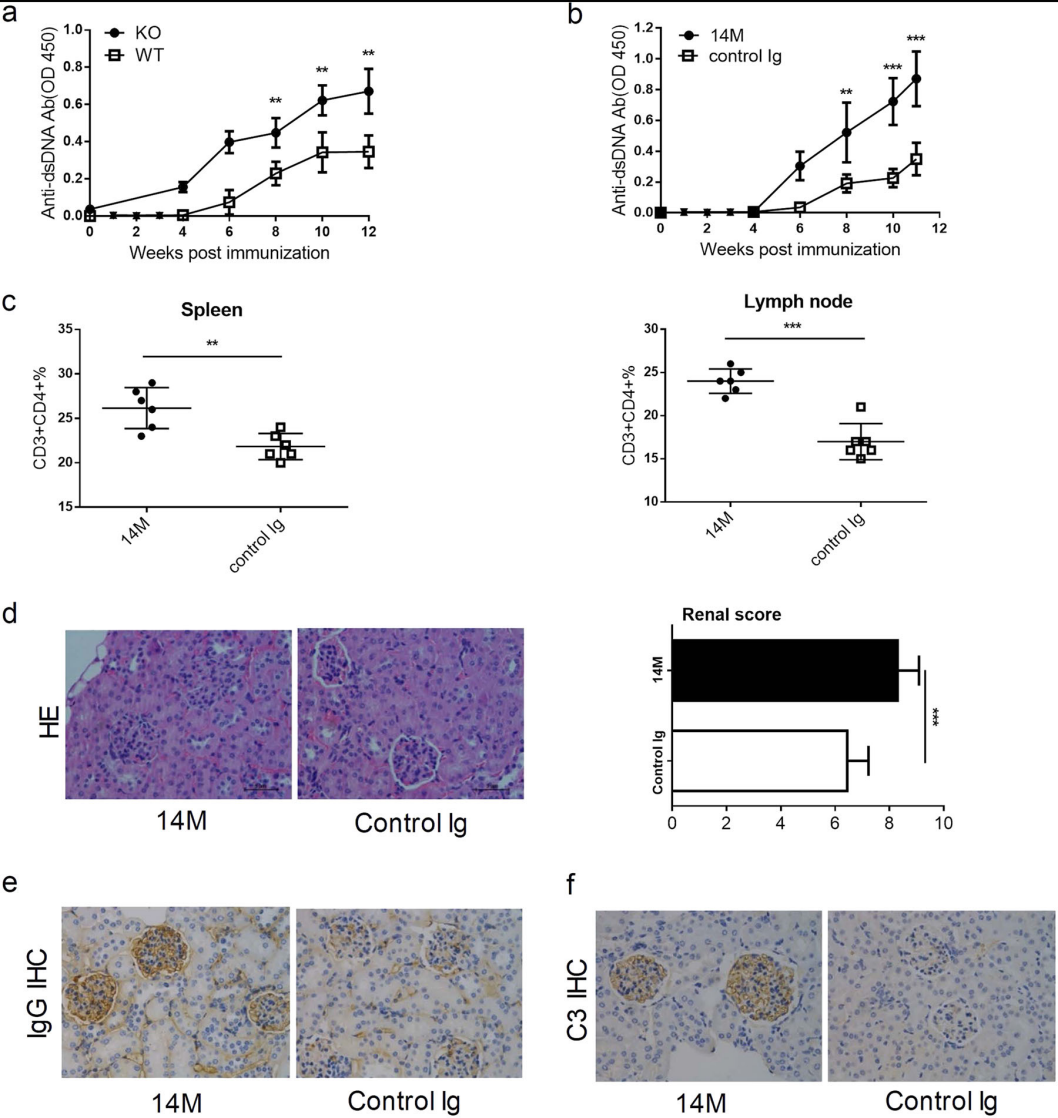
Intrinsic B7-H3 suppresses the production of anti-DNA Abs and renal inflammation in a SLE mouse model. **a** WT or B7-H3KO mice in groups of 5 were immunized with ALD-DNA (50ug/mouse) at 0, 2, 4 weeks. Sera from each mouse were collected at the indicated time points and anti-dsDNA Abs were analyzed by specific ELISA. **b** B6 mice in groups of 6 were immunized with ALD-DNA (50 μg/mouse) at 0,2,4 weeks and were treated i.p. with 14 M or control Ig weekly. Anti-dsDNA Abs in sera were analyzed at the indicated time by ELISA. **c**. Four weeks after three ALD-DNA immunizations, CD3+CD4+ T cells in spleen (left panel) and lymph nodes (right panel) were numerated by flow cytometry with specific mAb in mice treated with 14 M (filled circles) or control Ig (hollow squares). Each circle or square represents the result for each mouse. **d**–**f** Eight weeks after three ALD-DNA immunizations, mouse kidney sections were stained by hematoxylin and eosin (400×) (**d** left), the renal score of two groups (**d** right), IgG immunohistochemically staining (400×) (**e**), complement 3 immunohistochemically staining (400×) (**f**). Results are representatives of five mice

To evaluate possible role of B7-H3 in the production of autoantibodies and the pathogenesis of SLE, we first immunized B7-H3KO and WT B6 mice with activated lymphocyte-derived DNA (ALD-DNA). After the immunization, sera from mice were measured for the levels of anti-double stranded DNA antibodies (anti-dsDNA Abs) by specific ELISA. B7-H3KO mice had significantly higher levels of anti-dsDNA Abs than control wild-type mice in day 8, 10, and 12 (Fig. 1a), implicating that B7-H3 is inhibitory for the production of anti-dsDNA Abs in this model. We also tested the effect of a B7-H3 mAb (clone 14 M) in this model. Upon the immunization with ALD-DNA, WT B6 mice were treated with 14 M or control immunoglobulin (Ig) and 4 weeks later, the levels of anti-dsDNA Abs in sera were tested by specific ELISA. Similar to B7-H3KO mice, the level of anti-dsDNA Abs in 14M-treated wild-type mice was significantly increased compared with those treated with control Ig (Fig. 1b). Flow cytometry analysis showed that 14M-treated mice had significantly higher levels of CD3 + CD4+ T cells in both spleen (left panel) and lymph node (right panel) than those treated with control Ig (Fig. 1c). At week 12 after the immunization, we also evaluated kidney damage by tissue H&E staining, autoantibody deposition and complement 3 deposition assays. Remarkably aggravated glomerulonephritis and increased autoantibody/complement depositions were observed in 14M-treated mice than the control (Fig. 1d–f). We also verified the function of B7-H3 in B6 lpr/lpr-KO or B6 lpr/lpr –WT mice (Supplementary fig. 1a-d). The effect of 14 M in B6 lpr/lpr model were also tested (Supplementary fig. 1e-g). Therefore, ablation of B7-H3 by either genetic KO or antibody blockade enhanced autoantibody production accompanied with more severe kidney impairment. Our data support that intrinsic B7-H3 suppresses autoantibody production and disease progression in this SLE model.

### Immunization with DNA-pulsed DCs induced SLE syndrome

**Fig. 2:**
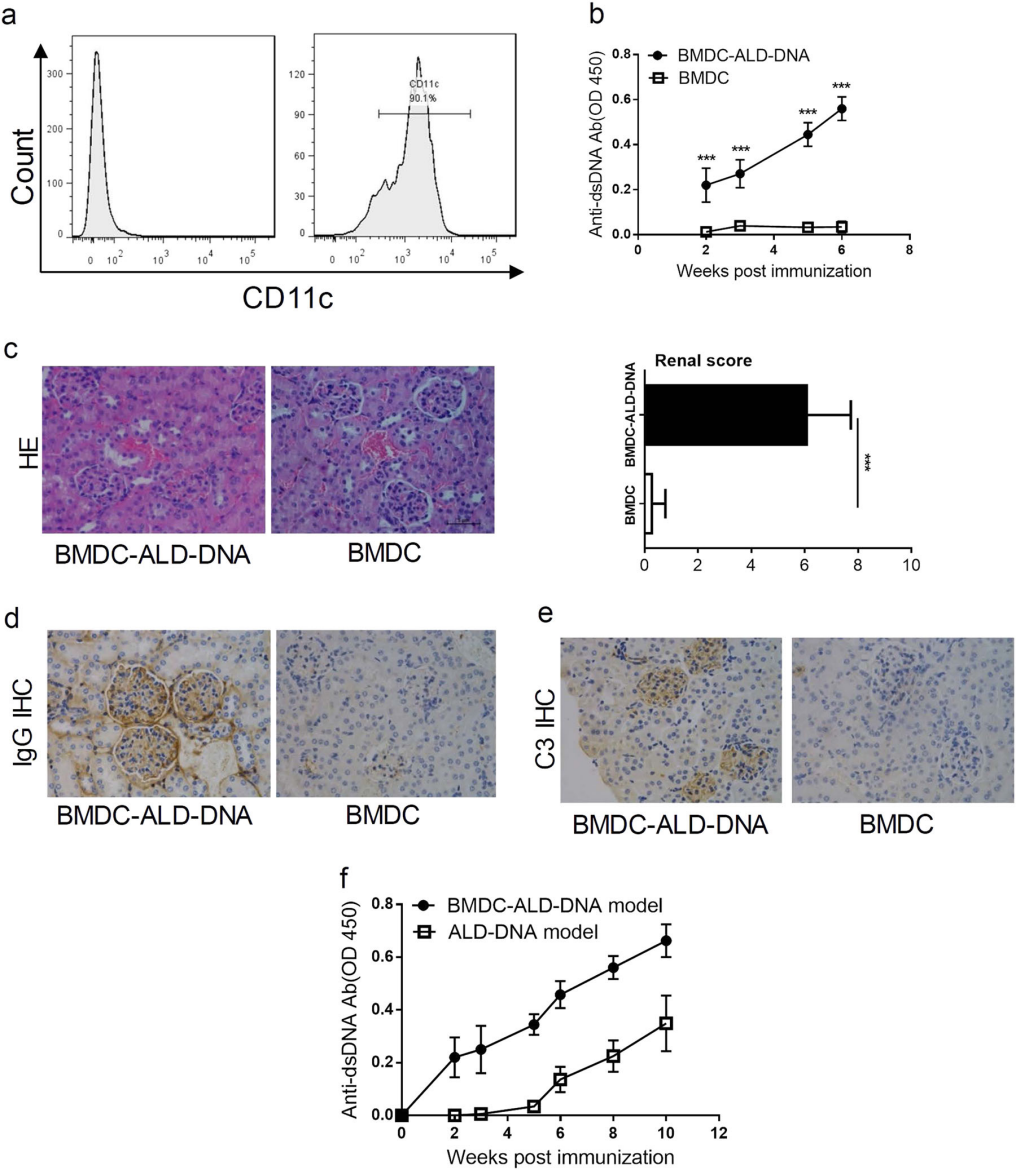
Immunization with DNA-pulsed dendritic cells induced SLE syndrome. **a** DCs were induced from bone marrow cells with GM-CSF/IL-4 and were stained with isotype-control antibody (left panel) or anti-CD11c-PE (right panel). **b** BMDC-ALD-DNA or BMDC at 10^6^ cells were intravenously injected into B6 mice. The serum from each mouse were collected and anti-dsDNA Abs were measured by specific ELISA. **c**–**e** Ten weeks after BMDC-ALD-DNA immunization, kidney sections were stained by hematoxylin and eosin (400×) (**c** left), the renal score of two groups(c right), IgG immunohistochemically staining (400×) (**d**), complement 3 (400×) (**e**), Results are representatives of five mice. **f** Mice were immunized with ALD-DNA (50 μg/mouse) or BMDC-ALD-DNA at 10^6^ cells by intravenous injections. The serum from each mouse were collected every two weeks and the anti-dsDNA Abs were detected by specific ELISA

To test the role of DCs in the induction of SLE syndrome in our model, we first generated DC from bone marrow cells by in vitro cytokine stimulation (Fig. 2a) and subsequently pulsed the BMDC with ALD-DNA for 12 h. Immunization of mice with BMDC-ALD-DNA led to the production of anti-dsDNA Abs in sera while immunization with BMDC without ALD-DNA did not induce anti-dsDNA Abs (Fig. 2b). Moreover, immunization with BMDC-ALD-DNA induced glomerulonephritis, autoantibody deposition and complement deposition whereas immunization with BMDC without DNA show normal kidney without significant autoantibody or complement deposition (Fig. 2c, e). Immunization with either ALD-DNA in adjuvants or BMDC-ALD-DNA induced high levels of anti-dsDNA Abs (Fig. 2f), indicating that ALD-DNA may be presented naturally by DC to stimulate anti-dsDNA Abs upon the immunization.

### The production of anti-DNA Abs is CD4+ T cell-dependent

**Fig. 3:**
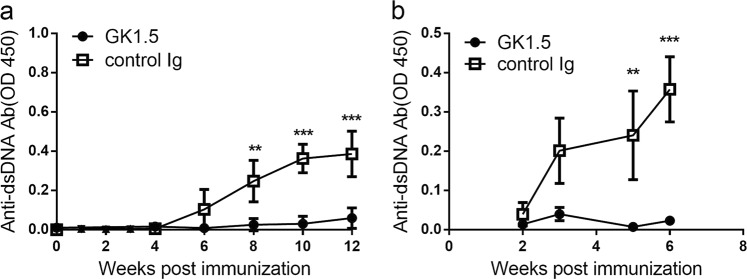
The production of anti-DNA Abs is CD4+ T cell-dependent. Mice at groups of five were depleted with CD4+ T cells by the injection of GK1.5 mAb (700 μg per mouse) i.p. inoculated 2 days before ALD-DNA (**a**) or BMDC-ALD-DNA (10^6^ cells) (**b**) immunization. The treatment of mice with GK1.5 were weekly. The serum from each mouse was collected and anti-dsDNA Abs were detected by specific ELISA

We next examined whether or not the production of anti-dsDNA Abs is dependent on CD4+ helper T cells. To test this, anti-CD4 mAb GK1.5 was injected into mice repeatedly to deplete CD4+ T in vivo after the immunization with ALD-DNA. Using this method, we demonstrated that the depletion of CD4+ T cells completely eliminated the production of anti-dsDNA Abs (Fig. 3a). Moreover, the depletion of CD4+ T cells also eliminated anti-dsDNA Abs production in the ALD-DNA-BMDC immunization model (Fig. 3b). Collectively, our results indicate that the production of anti-dsDNA Abs requires the help from CD4+ T cells.

### DC-associated B7-H3 suppresses the production of anti-DNA Abs and renal inflammation

**Fig. 4:**
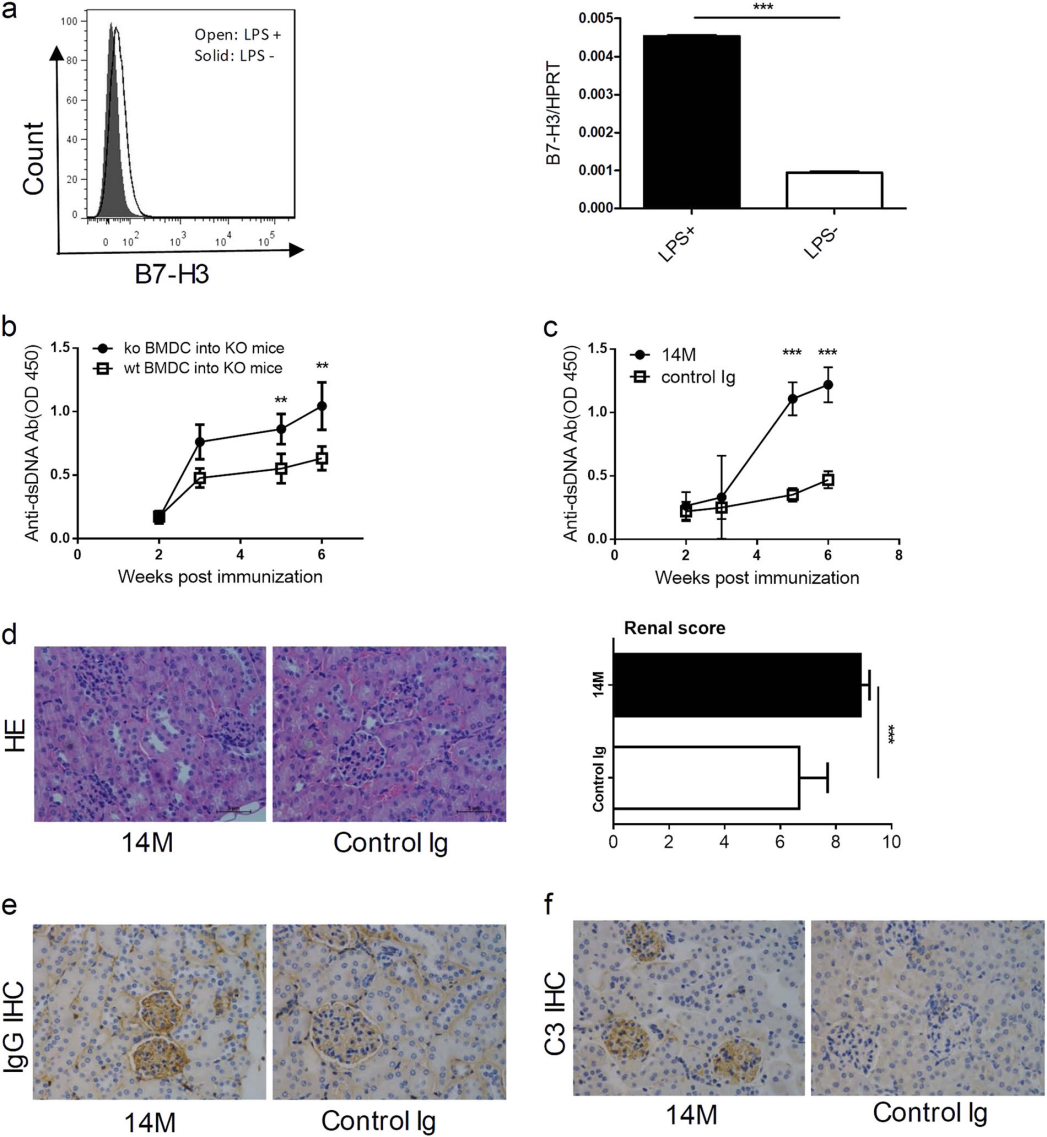
DC-associated B7-H3 suppresses the production of anti-DNA Abs and renal inflammation. **a** BMDC were stimulated with or without LPS and the expression of B7-H3 cell surface protein (left panel) or B7-H3 mRNA (right panel) were determined by flow cytometry with specific B7-H3 mAb (left) or qRT-PCR (right). **b** BMDC at 10^6^ cells from WT or B7-H3KO mice were pre-incubated with ALD-DNA(5ug/ml) and were intravenously injected into B7-H3KO mice for immunization. The serum was collected from each mouse every two weeks and the anti-dsDNA Abs were detected by specific ELISA. **c**. BMDC-ALD-DNA at 10^6^ cells were intravenously injected into B6 mice for immunization and subsequently treated with 14 M or control Ig weekly. The serum was collected from each mouse every two weeks and the anti-dsDNA Abs were detected by specific ELISA. **d**–**f** Ten weeks after BMDC-ALD-DNA immunization, kidney sections were stained by hematoxylin and eosin (400×) (**d** left), the renal score of two groups (**d** right), IgG immunohistochemically staining (400×) (**e**) and complement 3 immunohistochemically staining (400×) (**f**). Results are representatives of five mice

To evaluate the role of B7-H3 in DC-mediated induction of anti-DNA autoantibodies, we first examined the expression of B7-H3 by BMDC. As shown, while resting BMDC expressed very low levels of B7-H3 in both protein or mRNA, incubation of BMDC with LPS significantly upregulated its expression (Fig. 4a), indicating that B7-H3 is largely an inducible molecule on DC.

To test the role of DC-associated B7-H3 in the induction of anti-DNA autoantibodies, mice were immunized with DC from either WT or B7-H3KO mice. The KO-BMDC-ALD-DNA induced significantly higher levels of anti-dsDNA Abs than WT-BMDC-ALD-DNA (Fig. 4b). Consistent to this finding, the treatment by 14 M upon the immunization with WT-BMDC-ALD-DNA increased anti-DNA Abs production (Fig. 4c) as well as aggravated glomerulonephritis, increased autoantibody deposition and complement deposition (Fig. 4d–f). Our results indicate that DC-associated B7-H3 plays important role in the induction of anti-DNA Abs and inflammation.

### Blockade of B7-H3 enhances DC activation

**Fig. 5:**
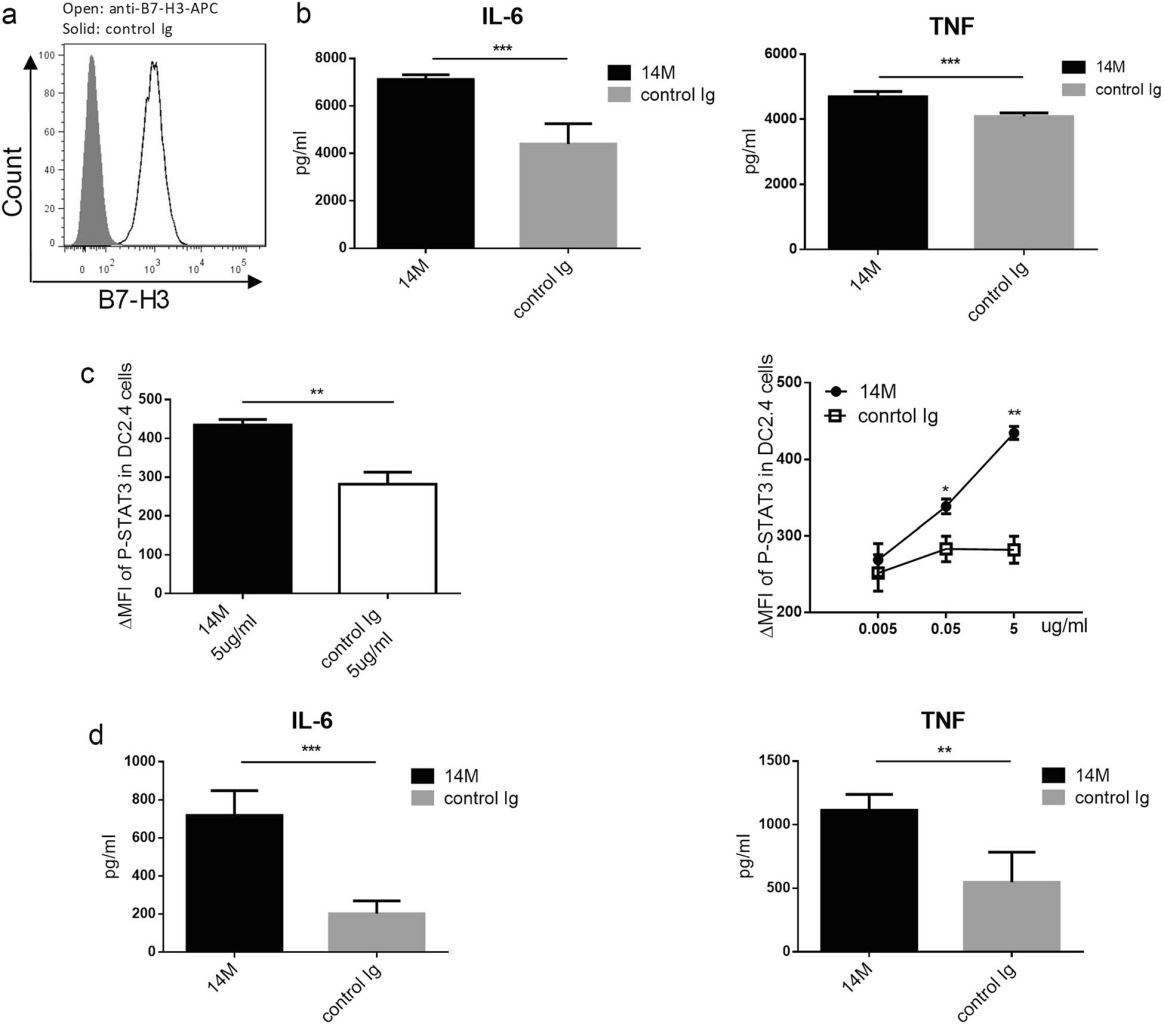
Blockade of B7-H3 enhances DC activation. **a** DC2.4 cells were stained with anti-B7-H3 mAb/APC (open) or control Ig (solid) and analyzed by flow cytometry. **b** DC2.4 cells at 5 × 10^5^ were cultured with LPS (1ug/ml) for 24 h and subsequently with 14 M or control Ig at 5ug/ml for 24 h. Supernatants were collected for cytokine CBA. **c** After stimulation of DC2.4 by 0.005, 0.05, and 5μg/ml doses of 14 M or control Ig for 24 h, cells were intracellularly staining with an anti-phosphorylated STAT3 antibody. **d** Freshly prepared BMDC at 5 × 10^5^ were cultured with LPS (1ug/ml) for 24 h and subsequently treated with plate-coated 14 M or control Ig at 5ug/ml for 24 h. Supernatants were collected for cytokine CBA

To determine the potential function of B7-H3 on DCs, an established cell line of DC (DC2.4)^23^ was used, which constitutively expressed high levels of B7-H3 as determined by flow cytometry after stained with anti-B7-H3 mAb (Fig. 5a). Interestingly, inclusion of 14 M in the culture significantly promoted the production of IL-6 and TNF compared with the control Ig-treated DCs (Fig. 5b). It is well known that IL-6 expression is closely linked with STAT3 activation^28^ and there is a positive feedback loop between IL-6 and STAT3 activation. We examined the levels of phosphorylation of STAT-3 (p-STAT-3) and showed that 14 M treatment significantly increased the level of p-STAT-3 in DC2.4 cells in a dose dependent fashion compared with control Ig (Fig. 5c). These results suggest that intrinsic B7-H3 on DC2.4 may suppress IL-6 and TNF secretion and STAT-3 phosphorylation. Similar results were also obtained using BMDC: inclusion of 14 M significantly enhanced the production of TNF and IL-6 from BMDC (Fig. 5d). Our findings thus indicate that blockade of B7-H3 promotes activation of DCs. Because DC2.4 is an established cell line and our findings implicate that 14 M blocks a putative receptor on DC to interact with B7-H3.

### B7-H3 agonist ameliorates inflammatory symptoms in the SLE mouse model

**Fig. 6:**
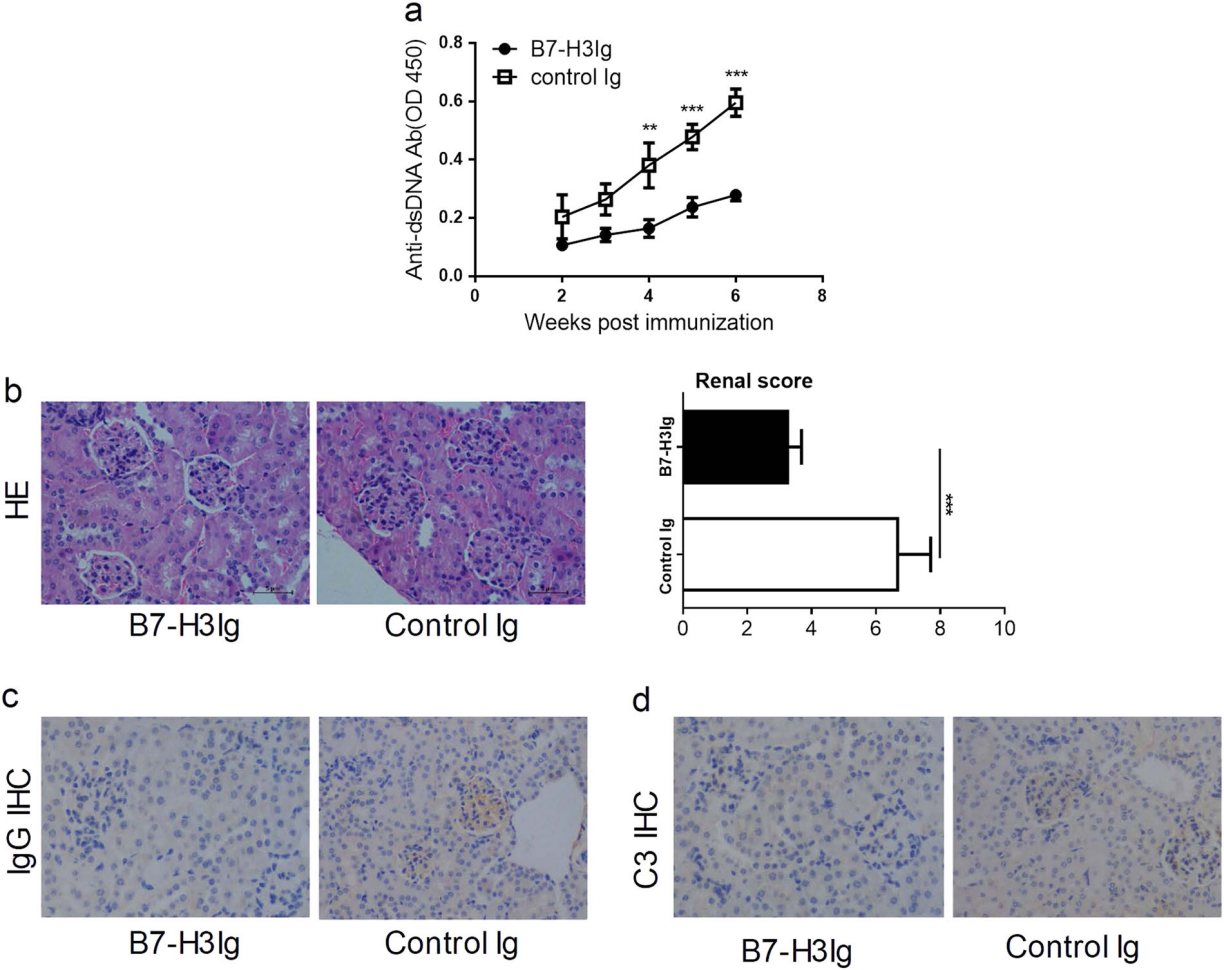
B7-H3 agonist ameliorates inflammatory symptoms in the SLE mouse model. **a** After the immunization with BMDC-ALD-DNA (10^6^), mice were treated with B7-H3 Ig or control Ig at 200 μg/0.5 ml weekly and the anti-dsDNA Abs in sera were assayed by specific ELISA. **b**–**d** Ten weeks after 10^6^ BMDC-ALD-DNA immunization, kidney sections were stained by hematoxylin and eosin (400×) (**b** left), the renal score of two groups(b right), IgG immunohistochemically staining (400×) (**c**) and complement 3 immunohistochemically staining (400×) (**d**). Results are representatives of five mice

Our data above suggest that endogenous B7-H3 is suppressive for the initiation and development of lupus-like symptoms in the SLE mouse model. We next determined whether or not enforced expression of recombinant B7-H3Ig, as a agonist, could relieve murine SLE symptoms. To test this, mice were treated with B7-H3Ig or control Ig after the immunization with BMDC-ALD-DNA. The mice that received B7-H3Ig showed a significant decrease in anti-dsDNA Abs production compared with control Ig groups (Fig. 6a). Remarkably alleviated glomerulonephritis, decreased autoantibody deposition and complement deposition were found in B7-H3Ig-treated SLE mice than control Ig treated SLE mice (Fig. 6b–d). However, mice were treated with B7-H3Ig after lupus injuries have occurred, the level of anti-dsDNA Abs in B7-H3Ig treatment was significant decrease compared with control Ig treatment (Supplementary fig. 2a) but renal score showed no difference (Supplementary fig. 2b) and the same degree of autoantibody deposition and complement deposition between two groups (Supplementary fig. 2c, d). Therefore, B7-H3Ig plays a more important in suppressing the progression of SLE disease at the early stage, especially in renal lesions. Our findings implicate a possible role of B7-H3 agonist as therapeutic agents for clinical SLE.

## Discussion

By ablation of B7-H3, either genetic deficiency or blocking mAb, our results indicate that B7-H3 may play an important role in the generation of anti-dsDNA Abs and subsequent promotion of renal inflammation in a SLE mouse model. Furthermore, we demonstrate that B7-H3 on DCs is required for the generation of anti-dsDNA Abs; while infusion of recombinant B7-H3Ig as agonist could suppress the production of anti-dsDNA Abs and ameliorate the disease progression in a mouse SLE model. Our findings implicate a role of B7-H3 pathway in the initiation and progression of human SLE.

DNA extracted from activated lymphocytes (ALD-DNA) was shown to induce anti-dsDNA Abs and subsequent promotion of SLE-like symptoms, including chronic inflammation in kidney^17,29,30^. DNA extracted from Con A-activated lymphocytes could trigger anti-dsDNA Abs production, leading to immune complex deposits in the kidney of syngeneic mice^31^. However, it is unclear how these DNA can immunize the host to generate anti-dsDNA Abs. In the present study we used BMDC which were coated with ALD-DNA, for the immunization and demonstrate their ability to trigger anti-dsDNA Ab in vivo. A widely used mouse strain for SLE model is MRL/lpr in which mice spontaneously develop autoantibodies and multiple organ impairments due to dysfunction of Fas/FasL cell death pathway^32^. B6 strain is not considered susceptible in general for the induction of autoantibodies and SLE. Our findings indicate that B6 mice could be induced to display SLE-like symptoms by the immunization with ALD-DNA and the ablation of B7-H3 promoted the progression of diseases. Currently clinical significance of these findings is yet to be explored. However, our findings suggest that decreased B7-H3 expression, especially on DCs, may be associated with more activated phenotypes of DCs and active SLE diseases and vice versa.

Constitutive expression of B7-H3 on a DC cell line, DC2.4, provides an opportunity to explore possible mechanisms of action. When 14 M was included in the culture of DC2.4 cells, higher levels of IL-6 and TNF than control were detected and this activation is accompanied with increased expression of STAT3-P (Fig. 5). Similar findings were also obtained using freshly isolated BMDC culture in which 14 M treatment could also promote IL-6 and TNF production (Fig. 5). Because in vivo experiments using the KO mice showed a role of B7-H3 in the suppression of autoantibody production, a major role of 14 M and B7-H3Ig fusion protein should be interpreted as blocking effect. At the present time, however, we could not exclude the possibility that 14 M may also have agonistic effect. It is possible that 14 M has both antagonist and agonist effect on B7-H3 while the antagonist effect is dominant in vivo. In this context, these in vitro findings suggest the presence of a B7-H3 inhibitory receptor on DCs. While molecular nature of B7-H3 receptor on DCs is yet to be identified, our findings have clinical implication since increased levels of IL-6 have been observed in SLE patients^33^ and its role in pathogenesis of SLE has been proposed^34,35^. Our findings are different from previously published studies suggesting the presence of B7-H3 receptor on T cells^36^.

Administer of recombinant B7-H3Ig could attenuate clinical symptoms of SLE in our mouse model (Fig. 6). However, B7-H3Ig treatment after lupus injuries have occurred, can only alleviate the production of anti-dsDNA Abs, but cannot reverse renal injury (Supplementary fig. 2). This finding is encouraging and indicates that B7-H3 could be potentially used for the treatment of SLE at the early stage. It is tempting to speculate that dimeric B7-H3Ig engages its putative receptor on DCs and subsequently suppresses the induction of CD4+ T cells so as to inhibit autoantibody production. Taken together, our study reveals a previously unknown suppressive mechanism of B7-H3 via DCs and proposes a possible approach for the treatment of human SLE.

## Supplementary information

supplementary figure legend

Supplementary figure 1

Supplementary figure 2
