# Supplementary material for: Dendritic cell-associated B7-H3 suppresses the production of autoantibodies and renal inflammation in a mouse model of systemic lupus erythematosus
Source: Cell Death Dis. 2019 May 21;10(6):393. doi: 10.1038/s41419-019-1623-0 (PMC6529467; doi:10.1038/s41419-019-1623-0)
Supplement: Supplementary file 1 — supplementary figure legend [file 41419_2019_1623_MOESM1_ESM.docx]

**Supplementary figure legends**

**Supplementary figure.1**

a. B6 lpr/lpr-KO and B6 lpr/lpr-WT mice were bled from retro-orbital sinus every 6 weeks and the serum was collected the anti-dsDNA Abs were detected by specific ELISA.

b, c and d. All mice were sacrificed at 24 weeks and kidneys sections were stained by hematoxylin and eosin (400x) (b left), the renal score of two groups(b right), IgG immunohistochemically staining (400x) (c) and complement 3 immunohistochemically staining (400x) (d). Results are representatives of six mice.

e. B6 lpr/lpr mice in groups of 6 were treated ip with 14M or control Ig weekly. Anti-dsDNA Abs in sera were analyzed at the indicated time by ELISA.

f, g, h. All mice were sacrificed at 24 weeks and kidneys sections were stained by hematoxylin and eosin (400x) (f left), the renal score of two groups (f right), IgG immunohistochemically staining (400x) (g) and complement 3 immunohistochemically staining (400x) (h). Results are representatives of six mice.

**Supplementary figure.2**

a. After the immunization with BMDC-ALD-DNA (10^6^), mice were treated with B7-H3 Ig or control Ig at 200ug/0.5ml at week 4 and treated with them weekly. The anti-dsDNA Abs in sera were assayed by specific ELISA.

b, c and d. Ten weeks after 10^6^ BMDC-ALD-DNA immunization, kidney sections were stained by hematoxylin and eosin (400x) (b left), the renal score of two groups(b right), IgG immunohistochemically staining (400x) (c) and complement 3 immunohistochemically staining (400x) (d). Results are representatives of six mice.
