# Supplementary figures and images for: Dendritic cell-associated B7-H3 suppresses the production of autoantibodies and renal inflammation in a mouse model of systemic lupus erythematosus
Source: Cell Death Dis. 2019 May 21;10(6):393. doi: 10.1038/s41419-019-1623-0 (PMC6529467; doi:10.1038/s41419-019-1623-0)

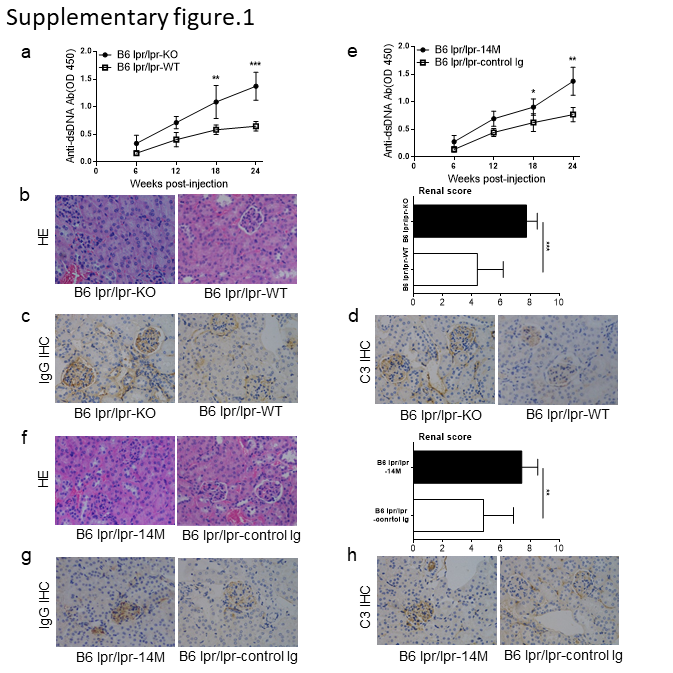

Supplement: Supplementary file 2 — Supplementary figure 1 [file 41419_2019_1623_MOESM2_ESM.tif]

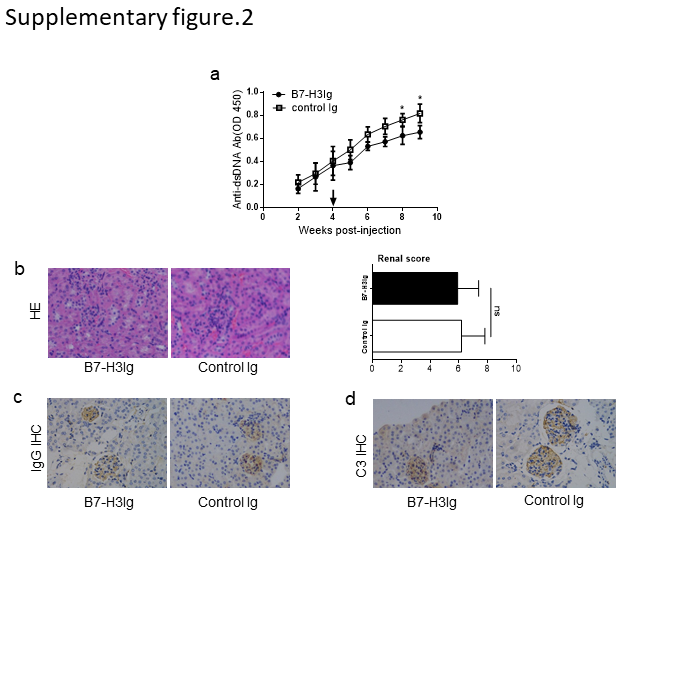

Supplement: Supplementary file 3 — Supplementary figure 2 [file 41419_2019_1623_MOESM3_ESM.tif]
